# Photosystem II-based biomimetic assembly for enhanced photosynthesis

**DOI:** 10.1093/nsr/nwab051

**Published:** 2021-03-30

**Authors:** Mingjun Xuan, Junbai Li

**Affiliations:** Beijing National Laboratory for Molecular Sciences (BNLMS), CAS Key Lab of Colloid, Interface and Chemical Thermodynamics, Institute of Chemistry, Chinese Academy of Sciences, Beijing 100190, China; Beijing National Laboratory for Molecular Sciences (BNLMS), CAS Key Lab of Colloid, Interface and Chemical Thermodynamics, Institute of Chemistry, Chinese Academy of Sciences, Beijing 100190, China; University of Chinese Academy of Sciences, Beijing 100049, China

**Keywords:** photosystem II, biomimetic assembly, photosynthesis, photoelectrobiological chemistry, artificial chloroplast

## Abstract

Photosystem II (PSII) is a fascinating photosynthesis-involved enzyme, participating in sunlight-harvest, water splitting, oxygen release, and proton/electron generation and transfer. Scientists have been inspired to couple PSII with synthetic hierarchical structures via biomimetic assembly, facilitating attainment of natural photosynthesis processes, such as photocatalytic water splitting, electron transfer and ATP synthesis, *in vivo*. In the past decade, there has been significant progress in PSII-based biomimetic systems, such as artificial chloroplasts and photoelectrochemical cells. The biomimetic assembly approach helps PSII gather functions and properties from synthetic materials, resulting in a complex with partly natural and partly synthetic components. PSII-based biomimetic assembly offers opportunities to forward semi-biohybrid research and synchronously inspire optimization of artificial light-harvest micro/nanodevices. This review summarizes recent studies on how PSII combines with artificial structures via molecular assembly and highlights PSII-based semi-natural biosystems which arise from synthetic parts and natural components. Moreover, we discuss the challenges and remaining problems for PSII-based systems and the outlook for their development and applications. We believe this topic provides inspiration for rational designs to develop biomimetic PSII-based semi-natural devices and further reveal the secrets of energy conversion within natural photosynthesis from the molecular level.

## Teaser

This review collects the recent contributions on PSII coupling with artificial structures via molecular assembly, and highlights how PSII-based semi-natural systems realize enhanced photosynthesis.

## INTRODUCTION

Studies into photosynthesis have formed the basis of Nobel Prize-winning research nine times: in 1915, 1930, 1937, 1938, 1961, 1965, 1978, 1988 and 1997 (Table 1) [1]. In particular, the Nobel Prize in Chemistry for 1988 was awarded to Johann Deisenhofer, Robert Huber and Hartmut Michel for ‘the determination of the three-dimensional structure of a photosynthetic reaction centre’. Their work systematically revealed the photosynthesis mechanism, providing an opportunity to advance research into photosynthesis with the aspect of bionic assembly.

**Table 1.** Photosynthesis-related Nobel Prize in Chemistry [1].

Year	Laureates	Rationale
1915	Richard Martin Willstätter	For his research on plant pigments, especially chlorophyll
1930	Hans Fischer	For his research into the constitution of haemin and chlorophyll and especially for his synthesis of haemin
1937	Norman Haworth	For his investigations on carbohydrates and vitamin C
	Paul Karrer	For his investigations on carotenoids, flavins and vitamins A and B2
1938	Richard Kuhn	For his work on carotenoids and vitamins
1961	Melvin Calvin	For his research on carbon dioxide assimilation in plants
1965	Robert Burns Woodward	For his outstanding achievements in the art of organic synthesis
1978	Peter D. Mitchell	For his contribution to the understanding of biological energy transfer through the formulation of the chemiosmotic theory
1988	Johann Deisenhofer, Robert Huber and Hartmut Michel	For the determination of the three-dimensional structure of a photosynthetic reaction centre
1997	Paul D. Boyer and John E. Walker	For their elucidation of the enzymatic mechanism underlying the synthesis of adenosine triphosphate (ATP)
	Jens C. Skou	For the first discovery of an ion-transporting enzyme, Na^+^, K^+^-ATPase

These brilliant achievements have encouraged scientists to pursue an efficient approach for light-harvest and utilization similar to natural photosynthesis [2,3]. Scientists have devoted much effort to the harvesting of light through production of new types of bionic structures or integration of naturally biological components into synthetic systems. A recent inspiration is imitation of natural photosynthesis in green plants, algae and cyanobacteria to convert light energy into chemical energy for driving life activities in organisms [4]. Photosynthesis implements efficient energy conversion through a light-dependent reaction and a dark reaction. Photosystem II (PSII) is a pigment-protein in the light reaction, responsible for the light-harvesting as well as water splitting to release O_2_, protons and electrons [5–8]. PSII presents robust operation in oxidizing ∼100 water molecules per second upon full sunlight, much higher than the oxidization rates of human-made catalysts [9]. Research into reassembly of PSII *in vitro* is promising, with respect to photocatalysis, biological solar cells and bionic photosynthesis. Recently, substantial developments have been made in PSII-based assemblies, PSII-mimicking hybrid systems and utilization of PSII-related products for energy conversion. Relative applications and explorations have been achieved based on construction of PSII-based biomimetic systems for photocatalytic water splitting [10,11], preparation of photobioelectrochemical cells [12] and ATPase synthesis [13].

As a protein complex, the protein binding sites of PSII can be coupled with synthetic materials allowing distribution of its photocatalytic property and products for conversion of light to chemical energy and photocurrent generation [14]. In early stage research, PSII was ‘wired’ to the electrode surface of photoelectrochemical cells (PEC) via entrapment in a matrix made of osmium-modified redox polymer to serve as an electron donor or photocatalyst [8]. In pursuit of high current generation, structural distribution of PSII has been applied for improvement of light-harvest and output of electrons or protons. Structural immobilization of PSII has evolved from two-dimensions (2D) to three-dimensions (3D). The hierarchical distribution of PSII has been used to maximize the efficient area of light absorbance and offer a high payload yield of PSII. Besides PEC systems, there have been applications of bionic systems, liposomes, polymeric multilayer film and porous particles to load or encapsulate PSII, aiming to use large surface areas to accumulate PSII [13,15,16]. For example, PSII was coassembled with ATPase in synthetic structures to reconstruct artificial chloroplast for ATP synthesis [13]. Also, photosensitive materials have been used, such as quantum dots, TiO_2_ and graphene [17–20], coupled with PSII to result in high rates of electron transfer and improve the light utilization range from UV light to visible light [21]. These life-like hybrid assemblies dramatically combine a natural portion and a synthetic portion, offering great incentives to develop artificial photosynthetic systems for light-harvest and energy utilization. This review focuses on recent progress in PSII-involved assemblies and PSII-biomimicking systems from a biological perspective and introduces how PSII couples with synthetic structures as a proton generator. Next, we turn to the reconstitution of semi-natural chloroplast and advanced biomimicking-chloroplast for *in vitro* photosynthesis study. We also discuss the current remaining issues and outlook for future challenges in this research field.

## PSII: STRUCTURE AND FUNCTION

**Figure 1.**
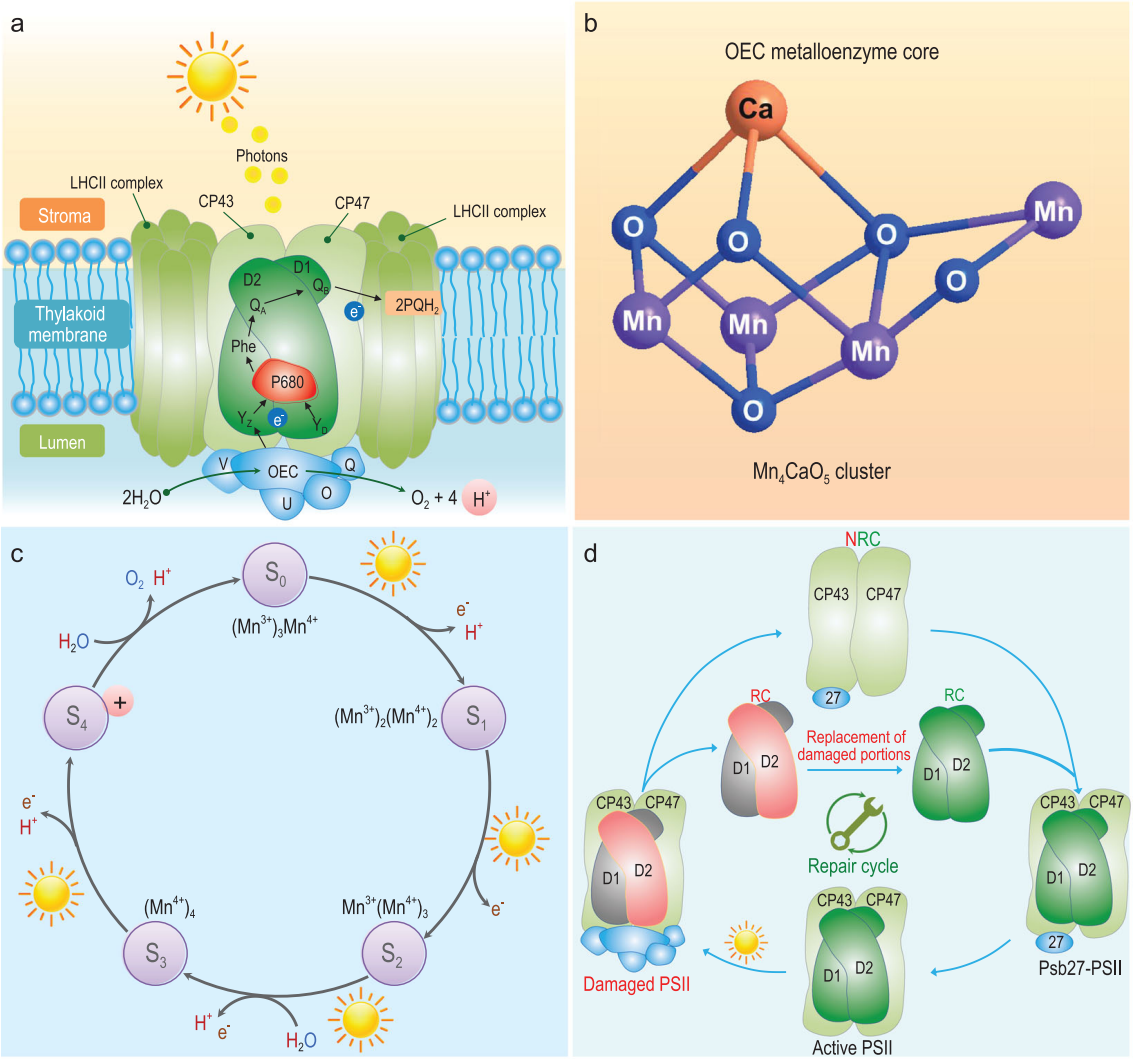
Schematic structure and function of PSII. (a) Structure of PSII. (b) Chemical structure of oxygen-evolving complex (OEC). (c) The current model of ‘kok’ cycle. (d) The ‘self-repair circle’ of PSII. Reprinted with permission from Ref. [26], Copyright 2019, National Academy of Sciences.

PSII is a type of pigment-protein complex consisting of multisubunits and cofactors, found extensively in the thylakoid membrane of plants, algae and cyanobacteria [22]. Together with photosystem I (PSI), cytochrome b6f complex and ATPase, PSII constructs the central portions of chloroplast for carrying out photosynthesis. There have been many significant advances in the understanding of PSII structure. PSII is composed of many subunits with a total molecular weight of 450–700 kDa, dependent on the source of organisms. Accessory subunits have been well studied, accompanying the structure resolution research of PSII from 3.8 to 1.9 Å using X-ray analysis [23]. The center position of PSII is the reaction center P680, named according to the combination with P (pigment: chlorophyll *a*) and a reactive and maximum absorption at 680 nm of light (Fig. 1a). P680 contains two transmembrane proteins (D1 and D2), which occur as a chlorophyll heterodimer to coordinate charge separation and the primary electron transport chain during photosynthesis. In the presence of light, P680 is changed to its excited state, P680^+^, to donate an electron [24]. Passing by pheophytin, the donated electron is transferred to the primary acceptor quinine (Q_A_), and subsequently to the secondary acceptor quinone (Q_B_), which provides PSI with electrons to produce the reducing power to convert CO_2_ to glucose. Subunits around D1 and D2 are chlorophyll-binding polypeptides, CP43 and CP47, with molecular weights of 43  and 47 kDa, respectively [25]. Both CP43 and CP47 are internal antenna systems of PSII that operate photon-harvest and transport photon energy to the reaction center, where oxygen-evolving complex (OEC) catalyzes the water splitting reaction. Several membrane extrinsic proteins (PsbO, PsbU, PsbV and PsbQ) surround OEC at the membrane–lumen interface [26]. Within OEC, a metalloenzyme core, composed of Mn_4_CaO_5_ cluster, efficiently processes water oxidation to produce O_2_, protons and electrons (Fig. 1b and c) [27].

The structure of PSII from eukaryotic organisms (higher plants and green algae) differs from that in photosynthesis-involved prokaryotic organisms (e.g. cyanobacteria). In higher plant and green algae, PSII often couples with peripheral light-harvesting complexes (LHCII) and minor chlorophyll-binding proteins composed of CP29 (29 kDa), CP26 (26 kDa) and CP24 (26 kDa), to form an advanced PSII–LHCII supercomplex [22]. LHCII gets together with CP43 and CP47 to absorb light and transfer photons to the reaction center of PSII for water splitting. Distinct from LHCII, the phycobilisome anchors to the surface of thylakoid membranes and plays the role of ‘antenna’ in harvesting light [28]. Apart from light-harvesting, another function of LHCII is to help PSII maintain regular operation by reducing any damage from strong light conditions in which the excess light energy is dissipated as heat [29].

The performance of PSII in cyanobacteria is often affected by the light intensity exceeding the saturating photosynthesis requirement, with a consequence of photosynthesis inhibition. Then, PSII begins to suffer from light-induced photodamage caused by the radicals and reactive O_2_ species accumulated through photochemistry of PSII [30]. In particular, proteins D1 and D2 are often destroyed in all proteins of PSII. A well-known process, the ‘PSII repair cycle’ has evolved against this unavoidable damage [31]. It is suggested that the repair process firstly operates *de novo* synthesis of damaged portions and partially disassembles PSII into its damaged parts (D1 or D2) and no-reaction-center (NRC) complex (Fig. 1d). After coordination of accessory protein Psb27, a new synthetic copy replaces the damaged proteins and reassembles PSII, so that PSII returns to its normal working state [26]. Although great signs of progress have been made in ‘PSII self-repair’, our understanding of how the repair cycle-related proteins work and synchronously coordinate with photosynthesis is not complete. Efforts are needed to address the remaining challenges in future research.

## PSII IN HIERARCHICAL STRUCTURES

**Figure 2.**
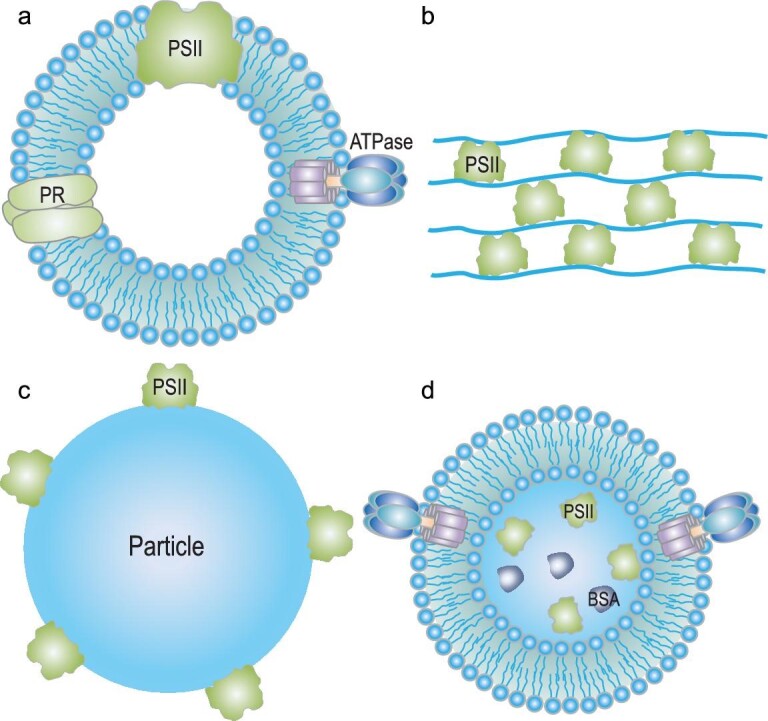
PSII-based biomimetic structures. (a) PSII couples with ATPase and proteorhodopsin (PR) into the membrane of a lipid vesicle. (b) PSII assembles in the polymeric multilayer films via a layer-by-layer assembly process. (c) The PSII-particle conjugates: PSII anchors on the surface of the particle. (d) The PSII-enriched particle with an ATPase-lipid coating.

The intrinsic function of PSII is to conduct the photocatalytic reaction of water and generate oxygen gas, protons and electrons. This effective process presents strong performance in light-harvesting and water splitting, beyond that currently possible in artificially synthetic systems. Thus, it would be very attractive to build a natural–artificial hybrid system, by integrating active PSII with synthetic materials to help optimize current light-responsive devices to offer new inspiration for design and development of biohybrid systems. At present, the study of PSII-based biomimetic systems is in its infancy. Sustained effort is still needed. Significant progress has been made in revealing the secrets of PSII structure and functions, facilitating active PSII extraction from plants and cyanobacteria. The bioactive PSII can assemble within various synthetic structures, such as lipid vesicles, porous microparticles and supramolecular structures (Fig. 2). With precise manipulation, these semi-natural biohybrid systems could regulate PSII payload yield and structural distribution. The light-harvest system and photocatalysis process in PSII could be moved into an *in vitro* environment, further providing PSII-related strategies toward energy conversion.

### PSII in the lipid membrane

Construction of lipid membranes is a successful way to imitate naturally bioactive membrane systems *in vitro* [32]. Many pioneering studies of biomimetic systems involve lipid membranes, such as protocells, artificial cells and synthetic organelles [33–36]. Lipid membranes offer a platform to combine a closed system and biocompatible characteristics, stabilizing guest biomolecules to form a bioactive membrane system. Their programmed components can also regulate the physicochemical properties of lipid layers. Generally, lipid membranes anchor PSII through a membrane fusion process. Tae Kyu Ahn, Kevin Kit Parker, Kwanwoo Shin and their coworkers report a type of giant lipid vesicle assembly with PSII, proteorhodopsin (PR) and ATPase, which forms a photosynthetic organelle for ATP synthesis (Fig. 2a) [15]. This biomimetic system achieves bottom-up reconstitution of active PSII *in vitro* and shows life-like functions. The emergence of microfluidic technology offers an emulsion strategy with which to assemble lipid-based multicompartmental materials. A recent study employed microfluidic fabrication to develop a mimic chloroplast droplet consisting of natural lipid membrane systems (PSII enriched-thylakoid membrane) and synthetic parts (16 enzymes of the crotonyl-CoA/ethylmalonyl-CoA/hydroxybutyryl-CoA (CETCH) cycle), which forms an innovative pathway for light-powered CO_2_ fixation [37]. Therefore, lipid membranes significantly match PSII and ensure their bioactivity for working *in vitro*.

Lipid membranes can be used to imitate a natural environment for PSII immobilization. Although natural active membrane systems are available, these can suffer from weak stability and insufficient mechanical property. Generally, lipid membranes are artificial systems in which the components are strictly made of amphiphilic compounds, resulting in the operation of PSII within a short time. Further optimizations could be made for PSII-lipid membranes. Transmembrane protein anchors could be used to increase their stability via increasing interaction between anchors and lipid molecules [38]. Cholesterol insertion has proven effective in facilitating and inhibiting the order state of phospholipids, and is also helpful in regulation of elastic performance and viscous property [39]. A previous study reported that polymersome can couple with natural proteins to overcome mechanical weakness by replacing traditional liposomes (e.g. ATPase) [33]. These methods promote PSII-lipid membrane systems attaining a stable state for the long term, useful for future exploration.

### PSII in multilayer structures

The assembly goal of PSII in artificial structures is to harvest light energy efficiently. As the payload yield and density of PSII are crucial, scientists have begun to focus on how to achieve a higher payload of PSII in synthetic structures. Layer-by-layer (LbL) assembly is a versatile tool to fabricate hierarchical polymeric multilayer materials [40–44]. LbL assembly involves electrostatic interaction, hydrogen bonding, covalent bond, metal coordination and van der Waals interaction [5]. Molecular building blocks can be deposited on templates to make multilayers, with the potential for many properties and functionalities to be obtained with PSII after assembly. The assembly occurs on shaped templates, such as planar surfaces, colloid particles and hole-enriched membranes, allowing for diversity in the assembled structures, such as multilayer films and capsules, and tubes. Polymeric multilayer materials formed by LbL assembly offer a great way to load many guest cargo types, such as proteins, DNA and nanoparticles [44–49]. Three approaches can be used in the loading process: incorporating multilayers, pre-loading to the templates and post-loading the assembled structures [50].

In 2002, Zhao and coworkers built up a PSII-based semi-biohybrid system to attain photocurrent generation in the presence of light (Fig. 2b) [51]. They used reaction center of PSII from *Rhodobacter sphaeroides* and positively charged poly(diallyldimethylammonium chloride) to assemble a protein multilayer film via electrostatic interaction. Moving forward with PSII assembly *in vitro*, several multilayered-PSII systems have been constructed by coassembly of PSII with synthetic materials in three-dimensions (3D). Since extraction of PSII from spinach, two approaches have been used to incorporate natural PSII into multilayer structures. In the first, PSII is considered as a negatively charged building block to assemble multilayer structures. PSII alternatively assembles with polyelectrolytes on the flat substrate and in the holes of cellulose acetate honeycomb membrane, allowing 3D distribution of the PSII [52]. The polyelectrolyte-derived material, a polyethyleneimine-stabilized reduced-graphene oxide (PEI-rGO), couples with PSII to form a PSII-enriched membrane [20]. In the second approach, PSII is entrapped in silica-based hydrogel and then injected into polyelectrolyte-modified porous polycarbonate (PC) film [53]. Such a compartmentalized sandwich structure contains a tubular array to improve the payload yield of PSII and achieve the hierarchical light-harvest. PSII in LbL assembled multilayer structures can achieve density regulation and 3D distribution.

In research into photobioelectrochemical cells, PSII-based layered structures have been used to obtain efficient electron transfer. PSII was ‘wired’ onto the electrode surface using an osmium-containing redox polymer based on poly(vinyl)imidazole. This thin film composed of polymer and PSII serves as a matrix to accept electrons [8]. In a further study, an electron transfer mediating layer was constructed, integrating PSI and PSII via crosslinking of polyvinyl pyridine/methyl pyridinium and cytochrome c [54]. Such layered structures offer great methods for assembly of advanced biomimetic platforms.

### PSII-particle conjugates

Nanoparticles have high surface area and flexibly engineered surfaces, which are attractive platforms for binding with proteins. In previous reports, gold and platinum nanoparticles were employed to conjugate with PSI via a covalent link, enhancing the function of H_2_ production [55]. Inspired by such PSI-nanoparticle conjugates, PSII has been conjugated with nanoparticles in attempts to improve active characteristics and performance. Takumi Noguchi and coworkers first functionalized the surface of gold nanoparticles (GNPs) with electron acceptor (CP47) of PSII from the thermophilic cyanobacterium *Thermosynechococcus elongatus*. In this system, a His6-tag was connected with the C-terminus of CP47 with nickel-nitrilotriacetic acid (Ni-NTA) modified GNPs. Consequently, the PSII was immobilized on the surface of GNPs with a diameter of 20 nm (Fig. 2c) [56]. A further study from the same group provides more details of the PSII–GNP complex [57]. Their results reveal that although PSII was quenched after conjugation with GNP, it showed enhanced durability compared with free PSII under strong-light illumination. Moreover, PSII can employ the inorganic particle photocatalyst (Ru/SrTiO_3_: Rh) and couple with an inorganic electron shuttle [Fe(CN)_6_^3–^/Fe(CN)_6_^4–^] as an electrochemical cell system for water splitting [58].

Generally, assembly occurs between PSII and particular systems in two ways. One is, as mentioned above, on the interfaces where PSII directly assembles on the surface of the particle. The second is by encapsulation of PSII into particles, which form a core-shell structure. Our group developed a type of PSII microparticle through a coprecipitation approach to encapsulate PSII and bovine serum albumin (BSA) together during the generation process of calcium carbonate (Fig. 2d) [13]. After glutaraldehyde (GA) crosslinking, subsequent removal of the CaCO_3_ template resulted in formation of PSII-enriched microparticles. This structure works as an artificial chloroplast by post-coating with an ATPase-incorporated lipid membrane and continuing the light-involved water splitting and ATP synthesis, demonstrating a conversion from light energy to chemical energy. At present, investigations into PSII-particle conjugates are rare. There is much promise in exploring how to conjugate more functional micro/nanoparticles with PSII. Future research could reveal many new features and functions of PSII-particle conjugates.

## MOLECULAR ASSEMBLY OF PSII-BASED SEMI-NATURAL BIOSYSTEMS

The combination of PSII and artificially synthetic structures is successful for making biohybrid assemblies. PSII attains a double match with synthetic systems in assembly of biomimetics to allow the performance of natural systems to be implanted into synthetic systems. Photosynthesis occurs in the thylakoid membrane in green plants, in which a photocatalytic reaction uses water and CO_2_ to produce O_2_, electrons, protons and glucose. PSII-related products are often regarded as benchmarks to evaluate the performance of PSII-based assemblies [9]. Although PSII presents a great state in synthetic systems, it would be attractive to pursue an efficient way to combine light-harvest and CO_2_ fixation as in a natural chloroplast. Molecular assembly is a fantastic way to construct or imitate biomimetic systems, which help us further understand efficient designs at the micro/nanoscale in nature [59–61]. Biomimetics are not limited to synthetic devices or systems and certain features or functions of biological entities; they can copy natural processes or activities.

Developments in biomolecular assembly have facilitated construction of PSII-based biomimetic systems. Several elegant and complex structures can incorporate versatile biological characters *in vitro*. Two purposes for PSII assembly *in vitro* demonstrate semi-artificial energy conversion devices, converting energy from light to electric power or biochemical energy. Many research groups have devoted their efforts to fabrication of PSII-based photoelectrochemical (PEC) cells [62–64]. PSII is used as a photoelectrode material and photocatalyst for water splitting and electron transfer. Besides assembly with synthetic materials, scientists can directly use biological components to build structured PSII assemblies. Natural components regulate better specific functions and higher efficiency than human-made systems, therefore, use of bioactive building blocks derived from nature together with PSII to assemble nature-like biomimetics is considerably attractive. The focus on the construction of advanced PSII-biohybrid systems integrates natural components into active biomimetic systems. Below, we describe the move towards a bionic aspect, and review the systems composed of PSII and natural components including PSI and ATPase. Additionally, an advanced biomimicking-chloroplast for enhanced ATP synthesis will be described.

### PSII-PSI assemblies as photoelectrochemical cells

**Figure 3.**
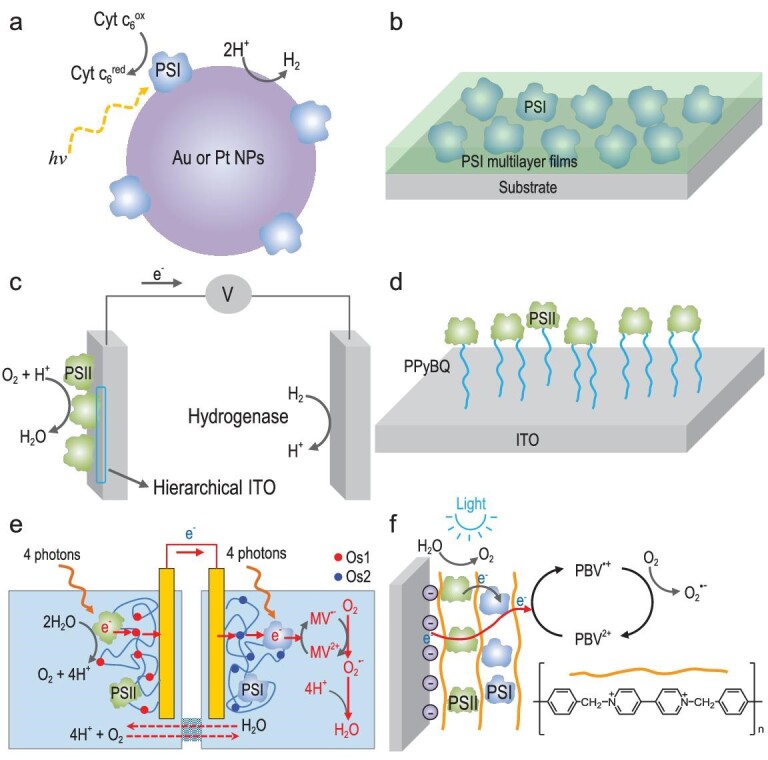
Electron transfer systems based on photosystem proteins range from PSI, PSII to PSI-PSII. (a) PSI-anchored Au or Pt nanoparticles for hydrogen production. (b) PSI-enriched multilayer films for photocurrent production. (c) PSII pairs with hydrogenase for photoelectrochemical water splitting. (d) PPyBQ nanowire-supported PSII-enriched membrane as a photoanode to generate photocurrent. (e) Full Z-scheme mimic strategy using PSI-based photocathode and a PSII-based photoanode to generate electrical energy. Reprinted with permission from Ref. [78], Copyright 2015, Wiley-VCH. (f) Bias-free water splitting system comprises multilayered PSII/PSI as PBV^2+^/PSI/PBQ/PSII. Reprinted with permission from Ref. [79], Copyright 2013, Wiley-VCH.

A popular way to achieve energy conversion from light to electric power is by use of semi-conductor materials [65]. With the continuous improvement in conversion efficiency, scientists began to note the potential of light-involved protein complexes, PSI and PSII, to implement light-harvest and light energy conversion to other energy types. PSI is a light-involved protein complex in the thylakoid membrane, one of two photosystem proteins [66]. PSI uses light energy to catalyze the reaction from plastocyanin to ferredoxin, while transferring electrons across the thylakoid membrane to reduce NADP^+^ to NADPH [67]. Although there is a specific connection between the activation of PSI and PSII in nature, PSI and PSII can be separately assembled into biomimetic systems dependent on their various purposes. For example, PSI is used to form nanoparticle systems via self-organized platinization or surface modification on noble nanoparticles for conducting hydrogen production (Fig. 3a) [68,69]. Because of its high reduction potential (−0.55 V) [66], multilayer films consisting of PSI matrix can be assembled onto several electrodes (e.g. indium tin oxide (ITO) conductive glass) as photoelectrochemically active redox centers, resulting in photocurrent production when merging into the electrochemical system (Fig. 3b) [70]. Moreover, PSI photobioelectrochemical electrode system is well developed via assembly of integrated photosystem I/glucose oxidase or glucose dehydrogenase photobioelectrochemical electrodes [71,72]. This system highlights the photonic wiring of the biocatalysts through PSI using glucose as fuel, which provides a way to assemble photobioelectrochemical solar cells with broad implications for solar energy conversion, bioelectrocatalysis and sensing.

PSII is the only enzyme that can use light to regulate a robust water oxidation process [15]. PSII conducts efficient light capture, takes the separation of photoexcited charges and assigns them to desired locations. Thus, PSII can be designed as a decorating material for anodes to donate electrons, resulting in photocurrent generation. Such photoanodes provide an innovative interface combining the characteristics of PSII and artificially inorganic materials. Erwin Reisner's group performed a series of pioneering works on PEC cells which take PSII as anode and PSII-hydrogenase electrode systems to generate the photocurrent with light exposure (Fig. 3c) [73,74]. The electrode surface-graft process enables redox polymers and synthetic porous materials, such as reduced graphene oxide [20], benzoquinone (BQ) doped PPy (Fig. 3d) [75] and porous TiO_2_ nanotube networks [19], to integrate successfully with PSII to fulfill the electron transfer. Apart from determination of material components, the intrinsic shape and structure are also crucial for construction of PSII-based electrodes. These strategies demonstrate a successful way in which to combine organic/inorganic current collector and PSII-based electron donors, leading to photocurrent densities in a diffusional mediator-free arrangement.

To promote integration of PSI and PSII, attempts have been made to ‘wire’ them to the surface of the electrode. In living photosynthetic organisms, water oxidation catalyzed by PSII generates electrons for the reductive processes driven by PSI, providing the inspiration for a coassembly of PSII and PSI, which achieves *in vitro* reconstruction of the electron transfer chain of the chloroplast [76]. Integrating PSII and PSI is challenging because PSII does not directly connect with PSI in nature. Dror Noy and coworkers reported a pioneering example of integrating PSII, b6f and PSI, connected by redox polymer, Os-complex modified hydrogel, and encapsulated into sol-gel glasses [77]. This platform used amphipathic quinone analogue 2,6-dichlorophenolindophenol (DCPIP) as the carrier to transfer electron passing through b6f from PSII to the reductive end-point of PSI. Another example conducted combination of PSII-based photoanode and PSI-based photocathode to a Z-Scheme mimic as a biophotovoltaic cell (Fig. 3e) [78]. This photoelectrochemical cell system took only water as fuel to implement electron generation at the PSII electrode. In the meantime, cytochrome c could be employed to couple PSII and PSI in assembling photobioelectrochemical cells [54]. However, current PSII-PSI PEC systems share a common drawback: a bias potential supply is essential to assist the system generating photocurrent. To address this issue, it would be necessary to fabricate some bias-free water splitting systems. Itamar Willner's group evolved construction of multilayered PSII/PSI as a PBV^2+^/PSI/PBQ/PSII system in which the potential was 0.0 V vs. Ag/AgCl (Fig. 3f) [79]. A recent approach coupled hydrogenase with the oriented PSI-modified electrode, allowing efficient electron transfer and optimizing a short-circuiting pathway [80]. A study employed a graphite-based cell system comprising quinone/hydroquinone and ferricyanide/ferrocyanide [81], respectively, which served as a potential supply of 12 V and drove a CdS-PSII-based PEC system for water splitting. Without the input of external potential bias, the conversion efficiency of solar-to-hydrogen can reach ∼0.34% in the presence of AM 1.5 G solar simulator irradiation (100 mW/cm^2^) [82]. Although this approach is highly specific to PSII-based PEC systems, it could be promising to apply this technology to a PSII/PSI photosystem in the future.

### PSII-ATPase systems as semi-artificial chloroplasts

ATP synthase (ATPase) is a transmembrane rotary protein, which accumulates in the thylakoid membrane and inner membrane of mitochondria. It is an energy-related protein that carries oxidative phosphorylation and conducts the conversion between chemical energy and mechanical works. In nature, the role of ATPase regulates proton flow from the biocatalytic photolysis of water by PSII. The generated protons stay in the chambers of the thylakoids. When the proton concentration reaches a high state, mechanical rotation of ATPase is triggered to synthesize adenosine triphosphate (ATP) [83]. This energetic molecular ATP can be used directly in the Calvin cycle, in which carbon fixation occurs to produce sugar.

The photosystem of the chloroplast is an intelligent system. Researchers have devoted effort to identifying and copying its working mechanism and natural design to optimize synthetic devices for ATP synthesis. In living photosynthetic organisms, light-responsive protons supplied by PSII intelligently regulate ATP synthesis. This coordinated action between ATPase and PSII inspired a bionic strategy to reconstruct artificial chloroplasts. Combination of PSII and ATPase *in vitro* could offer vital support to improve light-harvest and energy conversion in artificially synthetic devices. Moreover, reconstitution of such an intelligent system to perform natural photosynthesis *in vitro* would also be useful to help us understand photosynthesis mechanisms and develop new biomimetic materials.

ATPase successfully assembles with various synthetic materials and naturally active components [84]. A pioneering example in 2005 reported an imitation chloroplast using ATPase/bacteriorhodopsin (BR)-incorporated polymersomes for light-responsive ATP synthesis [33]. BR served as a proton pump to create a proton gradient for ATPase via a light-responsive BR photocycle, resulting in the ATP synthesis. Our group was also a pioneer in this research, contributing efforts to develop ATPase-based biomimetics [5]. In 2007, we achieved assembly of ATPase in the lipid shell of a hollow polymeric microcapsule in which ATP synthesis-storage was performed [85]. Subsequently, we developed a glucose-stimuli microcapsule to generate protons for ATPase synthesis in the presence of catalysis by glucose oxidase [86,87].

**Figure 4.**
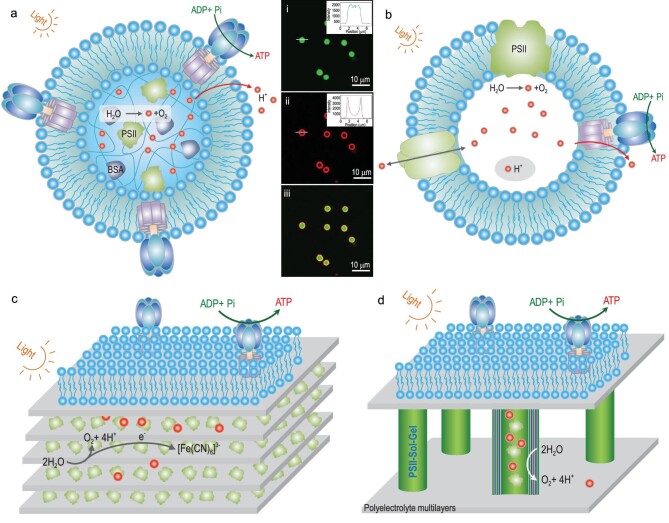
Semi-artificial chloroplast systems with the combination of PSII and ATPase for *in vitro* ATP synthesis. (a) An artificial chloroplast microparticle based on coassembly of PSII and ATPase for light-activated ATP synthesis. Confocal laser scanning microscope image of PSII-microparticle with ATPase-liposome coating: (i) chlorophylls in PSII (green light); (ii) Texas red-labeled proteoliposomes (red light); (iii) overlay image. Reprinted with permission from Ref. [13], Copyright 2016, American Chemical Society. (b) The artificial protocellular system is made of a giant vesicle containing ATPase and two photoconverters (plant-derived PSII and bacteria-derived PR). Red light facilitates PSII to generate proton for driving ATP synthesis, while green light impedes ATP synthesis by PR-depleting protons. (c) 3D distribution of PSII and ATPase in an artificial honeycomb multilayer to imitate natural thylakoid structure for light-activated ATP synthesis. (d) A polymeric structure with hierarchical and compartmentalized features for enhanced ATP synthesis.

Although such ATPase-based systems can perform partial functions of the chloroplasts, the ultimate target is to achieve chloroplast reconstitution *in vitro*. Integration of PSII and ATPase could facilitate this challenge. In 2016, our group reported a pioneering study of an artificial chloroplast via coassembly of PSII and ATPase (Fig. 4a) [13]. PSII and BSA get together, fixed inside CaCO_3_ microparticles via crosslinking of glutaric dialdehyde. After CaCO_3_ removal, an ATPase-proteoliposome was used to coat the surface of the PSII/BSA microparticles. This intelligent design yields morphology (shape and size) close to that of natural chloroplasts. When light illumination is applied, water oxidation and ATP synthesis happen as if occurring in natural chloroplasts. Another clever design worth highlighting involves an artificial protocellular system comprising a giant vesicle containing ATPase and two photoconverters (plant-derived PSII and bacteria-derived PR) (Fig. 4b) [15]. Independent optical activation of blue and red light-activated PSII and green light-activated PR enables ATP synthesis in a controlled way. The activation of PSII drives ATP synthesis using the generated protons, while the activation of PR blocks ATP synthesis through proton consumption, resulting in ‘on-demand’ ATP synthesis.

PSII-ATPase systems rely on light-harvesting to produce ATP. Light-absorbing performance and PSII-loading yield are essential factors to determine the conversion efficiency of PSII-ATPase from light to chemical energy. Structural optimization usually induces efficient light-harvest in semi-conductor solar cells. Our group successfully took hierarchical structures to achieve 3D distribution of PSII and ATPase via the LbL assembly process. We designed an artificial honeycomb multilayer to imitate natural thylakoid structure for light-activated ATP synthesis (Fig. 4c) [52]. PSII and positively charged polyethyleneimine were alternately assembled inside a porous cellulose acetate honeycomb membrane. By coating with ATPase-enriched lipid membrane, five 3D PSII-ATPase bilayers produce ATP with high efficiency over 70 μM (mg Chl^−1^) upon exposure to light illumination. Another work from our group described a polymeric structure with hierarchical and compartmentalized features (Fig. 4d) [53]. PSII is protectively encapsulated by silica-based hydrogel and subsequently serves as a filler to load into the inner chamber of PAH/PSS-based polycarbonate (PC) membrane. By sacrificing the PC membrane and ATPase-liposome coating, PSII-ATPase cylindrical arrays held by PAH/PSS scaffold are fabricated. The phosphorylation efficiency of this system increases 14 times compared with that of natural chloroplasts. Herein, compartmentalization and integration strategies for assembling PSII-ATPase systems demonstrate a biomimetic approach to reconstituting natural chloroplasts *in vitro* and performing photosynthetic processes.

Artificial chloroplasts constructed by PSII-ATP systems have versatile properties and high precision in manipulation. Importantly, this strategy regulates ATP synthesis with optical dynamic control and without close contact. In the meantime, biomolecular assembly maintains PSII-ATPase systems with high bioactivity and uses structural optimization to improve bioactive performance in non-biological circumstances. These artificial-biohybrid PSII-ATPase systems have great potential applications, ranging from investigation of cellular physiology to assembly of bioinspired materials.

### Advanced biomimicking-chloroplasts and PSII beyond natural chloroplast

Through improved understanding of natural chloroplast and its biological process, advances are being made into exploration and challenges facing PSII-based biomimetic systems. Light absorption is critical for the photosynthetic activity of PSII. In nature, the quantum efficiency is ∼100% during the light-harvest stage of PSII in the chloroplast [17]. However, the subsequent light energy utilization and conversion is relatively low efficiency. One way in which to address this issue would be to aid light absorption of PSII to extend to the UV light range rather than only focusing on visible light.

**Figure 5.**
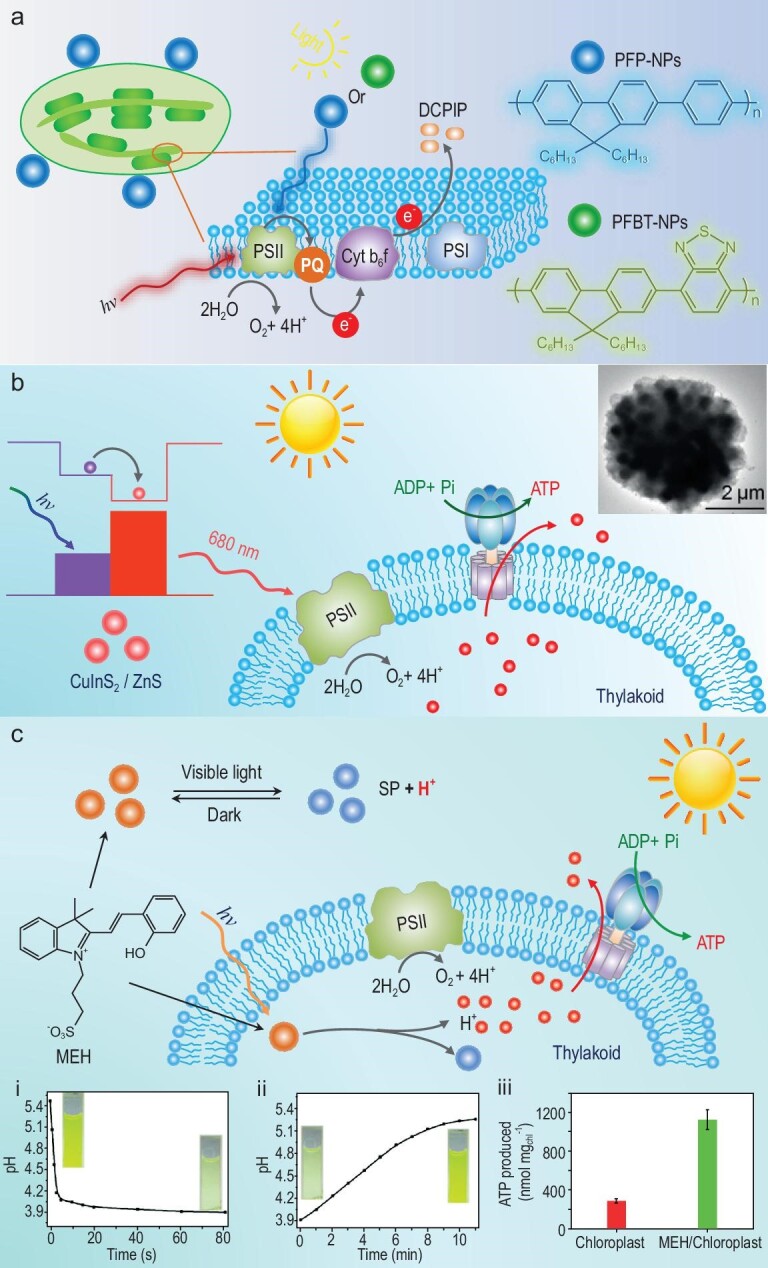
Biointerfacing engineering for biomimicking-chloroplasts through QDs modification. (a) Two fluorescent polymeric nanoparticles, PFP-NPs and PFBT-NPs, are used to coat on the surface of natural chloroplast to accelerate electron transfer via additional light absorption supply from UV light. (b) A couple of QDs, CuInS_2_/ZnS, is served as an optical converter that changes UV light to the 680 nm red light for optical absorption of PSII in chloroplast to enhance photophosphorylation. Reprinted with permission from Ref. [17], Copyright 2018, Wiley-VCH. (c) Long-lived photoacid molecule (merocyanine, MEH) into natural chloroplast for generating a stable supply of protons. The insets: (i) time-dependent pH of MEH; (ii) SP aqueous solution; (iii) ATP production. Reprinted with permission from Ref. [90], Copyright 2017, Wiley-VCH.

Scientists have identified a direct way to decorate natural chloroplast with light-absorbing materials to overcome the limitation of absorption in visible light. An interesting study reports a promising combination of chloroplast and light-harvesting materials, which improves the absorption of PSII in the range of UV light [21]. Two fluorescent polymers, poly[2,7-(9,9-dihexylfluorene)-co-alt-p-phenylene] (PFP) and poly[(9,9-dioctylfluorenyl-2,7-diyl)-co-(1,4-benzo-{2,1′,3}-thiadiazole)] (PFBT), are used to prepare conjugated polymer nanoparticles (CPNs), which coat the surface of natural chloroplast (Fig. 5a). CPNs are used as an optical amplifier to accelerate electron transfer and enable photosynthesis beyond natural chloroplasts via broad light absorption, including UV light. Our group developed similar strategies which directly mimic chloroplasts with light-responsive materials [17,58]. A natural biohybrid system comprises quantum dot (QD)-modified chloroplasts with a large Stokes shift [17]. In this system, a couple of QDs, CuInS_2_/ZnS QDs, serve as an optical converter to change UV light to the 680 nm red light that matches the maximum absorption of PSII in the chloroplast (Fig. 5b). Additional light supply is created by QDs to give higher efficiency to split water than pristine chloroplasts. In the meantime, a large proton concentration gradient generates across the thylakoid membrane, which drives enhanced ATPase synthesis. In particular, the photophosphorylation efficiency in QDs-chloroplast increases to 2.3 times that of natural chloroplast. This strategy successfully optimizes the utilization of UV light in a PSII-based electrode system. CdTe QDs facilitate the electrode made of PSII-enriched polyelectrolyte multilayers to generate a high photocurrent yield because of the increased electron density during the water splitting of PSII. A similar strategy can be applied in multilayered PEC systems. PSII couples with QDs to convert UV light to visible light, resulting in photocurrent enhanced output (Fig. 5c) [88]. Also, QDs offer a light source based on the large Stokes shift and could take the role of electron transfer. A recent study employed QDs to replace PSI’s electron transfer, enabling a high open-circuit voltage in a Z-scheme photobioelectrochemical cell system [89].

Decoration of natural chloroplasts is not the only way to improve their performance. Increased proton supply could achieve an enhanced photosynthetic process. ATP production is a ‘benchmark’ for PSII-based biomimetics and artificial chloroplast. We encapsulated a long-lived photoacid molecule (merocyanine, MEH) into a natural chloroplast to generate a stable supply of protons [90]. Upon illumination with sunlight, MEH triggers a fast intramolecular photoreaction to produce protons. The resulting strongly enhanced proton gradient improves the photophosphorylation efficiency, with 3.9 times the ATP production achieved using a MEH-encapsulated chloroplast compared with a natural chloroplast.

The strategies above offer promising approaches for mimicking natural chloroplasts, inspiring supramolecular assembly and synthetic biochemistry to construct artificial chloroplasts. Our group synthesized a type of multicompartmental silica nanoparticles with hierarchical structures that encapsulate photoacid generators and ATPase-liposomes, which serve as a biomimetic chloroplast [91]. With light illumination, photoacid generators create a proton gradient to drive ATP synthesis. A report described giant unilamellar vesicle (GUV)-based artificial cells encapsulating natural thylakoid to mimic chloroplasts [92]. As the partial processes of photosynthesis, carbon dioxide (CO_2_) capture and NADPH were implanted to perform in the artificial hybrid system. Electron transfer in the GUV system can be inhibited by 3-(3,4-dichlorophenyl)-1,1-dimethylurea and heavy metal ions (Hg^2+^, Cu^2+^, Cd^2+^, Pb^2+^, and Zn^2+^) during the photosynthesis process. An artificial thylakoid was constructed by decorating cadmium sulfide CdS QDs in the inner wall of protamine-titania (PTi) microcapsules. This system carried out photobiocoupled reduction of CO_2_ and NADH regeneration via formate dehydrogenase and formate/formaldehyde/alcohol dehydrogenase complex [93]. Apart from these above systems, some biomolecules can be employed to assemble biomimetic structures for mimicking chloroplasts. As an example, it was reported that enzyme-triggered covalent assembly of Cricoid stable protein can form one (SP1) system, incorporating 1D nanotubes and 2D nanosheets via a controlling Tyr site of SP1. This protein sheet system mimics the layered structure of natural thylakoid. By integrating with QDs, this system can perform the FRET phenomenon to carry out chlorophyll-like light-harvest [94].

At present, although development of artificial chloroplasts is in its infancy, investigation into this field offers great inspirations to pursue efficient solar energy harvest and conversion. In the meantime, artificial chloroplast systems can compensate for drawbacks (e.g. biological stability) shared by natural chloroplast-based biomimicking systems and PSII–ATPase based natural-artificial systems. Therefore, there should be continued to focus on performance optimization of artificial chloroplasts, to facilitate new features and functions of PSII-based biomimetics.

## CONCLUSION AND OUTLOOK

PSII is a fantastic, natural, light-involved energetic enzyme that lives extensively in green plants, algae and cyanobacteria. One appealing aspect of PSII is its ability to assemble with synthetic materials and natural components *in vitro*, enabling energy conversion from light to photocurrent and chemical energy. Although investigation of PSII-based systems is in the early stages, pioneering studies highlighted in this review have proven that the concept and potential of a semi-natural strategy are promising. Use of a bioinspired assembly will drive forward investigation into PSII-based systems. The assembly of PSII-based semi-biohybrid systems successfully moves part-natural photosynthesis into *in vitro* environments. PSII offers binding sites for coupling guest agents via protein-chemical modification approaches, which facilitates functionalization. PSII collects versatile synthetic materials to achieve high operation, promoted electron transfer, enhanced ATP synthesis and UV light-absorption. 3D distribution of PSII allows structural manipulation for improving light-harvest. Therefore, the current understanding of natural PSII is helping us to unveil the evolution secrets of efficient light-harvest.

The fast development of PSII-based systems has drawn researchers’ attention from the field of electrochemical cells and photocatalysis. PSII is promising for the exploitation of potential applications. It is regarded as the electrode component to catalyze water splitting and produce electrons, thus attaining a period of semi-natural artificial photoelectrochemistry. Moreover, the past decade's efforts to combine PSII and natural components permit a novel pathway for light transfer to bioenergy. This strategy provides an opportunity to make new organelle types, which could advance studies on direct assembly of natural bioactive components. Apart from such direct assembly with synthetic materials, PSII-based systems have inspired artificial chloroplasts, challenging and moving them beyond the natural design.

Although reassembly of natural PSII in hybrid systems has been successful, it shares the typical drawbacks of general protein species in terms of stability, durability and biological activity. Also, we should not ignore environmental restrictions for applying PSII-based systems. In particular, inactivation of biological components of PSII is a problem. Suggested solutions, such as protective coating and biofriendly assembly process, could help overcome the low operation of PSII *in vivo* caused by environmental factors. The mimicking technique for engineering PSII, thylakoid or chloroplast is an excellent approach to prolong stability, optimize performance and exploit applications. Another challenge is the relatively low conversion efficiency for subsequent utilization; only 3–4% of the total sunlight energy is converted into stored free energy during photosynthesis [95]. This unexpected behavior does not match with its excellent quantum efficiency for light-harvest. Even though QDs achieve conversion from UV light into visible light for direct utilization, it would be desirable to figure out the mechanism and intrinsically improve the conversion efficiency. These facts mean the field of PSII-based biomimetic assembly remains at the stage of theoretical research and laboratory exploration. That is a long way to go to achieve commercial application.

Looking to the future, PSII-based bioinspired systems are up-and-coming and still have unknown potential. Among the products of PSII, electrons and protons are involved in current studies and applications [96]. However, investigation into oxygen is lacking, thus it is expected this will be highlighted in future research. Also, intrinsic features of PSII, such as self-repair and smart ordination upon exposure to intense light, would be attractive to apply to design and development of innovative light-responsive materials or macroscopic materials (e.g. artificial leaf) [97]. The replacement strategies and non-biomaterial-based approaches are promising in development of light-involved materials [98,99]. For example, QDs could be used to replace PSI or polymeric donor-acceptor light-harvester, in construction of the electron transfer chain [89,100]. Some natural proteins may also have utility in this area. LHCII is worthy of note in the study of photovoltaic cells because of its remarkable property of light-harvest [101–103]. Further investigation of LHCII could be considered to improve the photocurrent density and production yield of artificial chloroplast systems or natural chloroplast-mimicking systems, perhaps even to develop LHCII-based light nanodevices.

## Supplementary Material

nwab051_Supplemental_File
