# Supplementary material for: Photosystem II-based biomimetic assembly for enhanced photosynthesis
Source: Natl Sci Rev. 2021 Mar 30;8(8):nwab051. doi: 10.1093/nsr/nwab051 (PMC8363332; doi:10.1093/nsr/nwab051)
Supplement: nwab051_Supplemental_File [file nwab051_supplemental_file.docx]

This review collects the recent contributions on PSII coupling with artificial structures via molecular assembly, and highlights how PSII-based semi-natural systems realize enhanced photosynthesis.
